# The Book World of Medicine and Science

**Published:** 1902-07-26

**Authors:** 


### THE BOOK WORLD OF MEDICINE AND SCIENCE.

ORTHOPÆDIC SURGERY: A HANDBOOK. By CHARLES BELL KEETLEY. (London: Smith, Elder and Co. Pp. 539. 8vo. Price 15s.)

MR. KEETLEY is so well-known as a surgeon and as an author, that it is with pleasurable feelings of anticipation we turn to a new volume from his pen. In the present work Mr. Keetley has incorporated much of his thought and practice during the last 20 years or so, and as a record of ripe experience it is therefore valuable. But it suffers from inherent defects which may perhaps limit its due influence. We allude particularly to defects of forms and arrangement; expecting from Mr. Keetley better things than that ,after an excellent introductory chapter on classification, he should lead us from Genu-Valgum to Rickets in general, and that a chapter on hysteria should be sandwiched between others on Equino-varus and wry neck. The book is, in fact, a collection of essays rather than a systematic treatise, and its value lies in its wealth of practical teaching rather than its pathological disquisitions. The second chapter—that on Genu Valgum—shows Mr. Keetley at his best. It is practical and definitive. The treatment of severe cases in adults and adolescents is summed up in two words—Macewen's osteotomy. But in slight cases and in early life there are alternatives which may be tried for a time. The next chapter — on Rickets — is less happy. We do not think Mr. Keetley exaggerates when he says that of all the causes of deformities the most important and the most extensive is rickets; but we demur when he propounds the hypothesis that rickets is a definite disease due to infection from without "like tubercle and syphilis." It is so much easier to predicate a microbe than to confess ignorance; but it is also far less scientific. Nor can we agree that we must "beware of eating or recommending any raw food whatever" in cases of rickets. But we are glad to recognise the force with which Mr. Keetley warns surgeons who are attempting to cure bowed legs and curved tibiæ lest they concentrate their attention on local appliances and neglect treatment of the rickets itself. Mr. Keetley's remarks on Infantile Paralysis are optimistic and furnish many useful hints on matters which surgeons are apt to forget, for, as the author says, little patients whose diseases are on the borderland between medicine and surgery sometimes slip between two stools. Mr. Keetley does not believe in the "muscular weakness" theory of pes planus, and is less positive than is his wont in dealing with talipes varus and equino-varus, conditions the etiology of whsch he says "is still unsettled and an affair of more or less well-grounded hypothesis." "Hysteria in relation to orthopædics" is the subject of a separate and not very powerful essay, from which there are some notable omissions—in particular Brodie's sign of hysterical joints. But there are some pungent criticisms of "gymnastic teachers," and those who cure hysterical spine by "movement exercises," which deserve attention.

A TEXT-BOOK OF PHYSICS, WITH SECTIONS ON THE APPLICATION OF PHYSICS TO PHYSIOLOGY AND MEDICINE. By R. A. LEHFELDT, D.Sc. (London: Edward Arnold. Price 6s.)

THIS is undoubtedly a good book, and will be found of great assistance by those who wish to obtain a proper grasp of the subject without plunging too deeply into mathematics. The whole matter is treated scientifically with logical progression from the more simple to the more complex, and useful cross references are given by aid of which it is easy to hark back for further information if one overruns one's knowledge. Beginning with a chapter on general physics which covers the ground occupied by the "mechanics" of the older examinations the author takes us in successive chapters through the subjects of heat, the properties of fluids, properties of solids, sound, chemistry, electric currents, electro-magnetism, electrostatics and light. Throughout the book the subjects are treated on what may be termed the experimental method, constant reference being made to the experiments by which the facts are to be demonstrated, as in the class-room; and to anyone who starts with a moderate knowledge of algebra and geometry everything is made most admirably clear. As showing the position of the book in the curriculum we may refer to the preface in which the author states that it is based on his lectures for the intermediate examination in science at London University. There is more in its pages than is required for the first conjoint examination, but by an arrangement of large and small type, the student is enabled to put on one side the more advanced parts.

SMALL-POX: ITS PREVENTION, TREATMENT, AND HISTORY. (London: Florence White. 1902. Price 6d.)

THE object of this book is, the compiler tells us, practical, not polemical: statements of fact are made, and the reader is to form his own conclusions. Let us therefore examine the "facts" put forward. On page 1 we are told "that it (small-pox) is just as infectious to adults as to children; that age is no protection whatever." We learn "that small-pox is caused by the reception into the blood of a microbe" and has a "characteristic greasy smell." The first "fact" is untrue; the second "fact" is unproved, and the third "fact" conveys no meaning to us. Proceeding, we note that the compiler fails to distinguish between small-pox with an early hæmorrhagic or purpuric rash and confluent small-pox with hæmorrhages into the pustules and from the mucous membranes. Chapter III. is devoted to a discussion on "How Small-pox Spreads," and is perhaps best characterised by saying that it alludes to every factor in the spread of small-pox except the absence of protection by vaccination of the infected population. Chapters IV., V., and VI., on "Vaccination," "The History of Small-pox," and "The Present Epidemic," are ingenious but also disingenuous. There is much show of impartiality. Great parade is made of the legislative enactments referring to vaccination, and great play is made with highly coloured word-pictures of the insanitary and overcrowded slum districts of London, in which small-pox has made the greatest havoc. But the compiler does not state—as he or she well might—that the revaccinated slum-dweller, though a *rara avis*, usually escapes small-pox, while his dirty, dissolute, and drunken neighbours (who, forming as they do one of the least intelligent portions of the community, form also one of the least vaccinated), go to crowd the hospital ships and camps of the Metropolitan Asylums Board.

